# Nitrous oxide fluxes and soil nitrogen contents over eight years in four cropping systems designed to meet both environmental and production goals: A French field nitrogen data set

**DOI:** 10.1016/j.dib.2021.107303

**Published:** 2021-08-16

**Authors:** Caroline Colnenne-David, Gilles Grandeau, Marie-Hélène Jeuffroy, Thierry Doré

**Affiliations:** Université Paris-Saclay, Inrae, AgroParisTech, Agronomie, Thiverval-Grignon 78850, France

**Keywords:** Nitrous oxide fluxes, Soil ammonia content, Soil nitrate content, Aboveground plant nitrogen content, Yield, Long-term field trial, Cropping system, Agricultural practices

## Abstract

With the development of agroecosystem approaches, new cropping systems have to be designed to deliver multiple ecosystem services. In this context, we assessed four innovative cropping systems, designed to reach multiple environmental and production goals, in a long-term field experiment (2009–2020) at Grignon (France, N 48.84°, E 1.95°). A wide range of measurements were made, for nutrient cycles and organic matter in particular, for an analysis of interactions occurring during the emissions of greenhouse gases. We focus here on nitrogen (N) data collected over eight years (2009–2016). The data include: nitrous oxide fluxes (N_2_O), soil N contents (NO_3_^−^ and NH_4_^+^), aboveground plant N content and biomass at maturity, yield, agricultural practices including N spreading, and climate. The four systems differ in terms of tillage practices, N inputs, and species, which is likely to affect soil N. Field data were collected and N_2_O fluxes were calculated. These original new cropping systems are innovating, resulting in new combinations of agricultural practices. The data obtained could be used to improve models for parameterization and validation, and to increase the predictive accuracy of models of N losses in original conditions.

## Specification Table

**Table:** 

Subject	Agronomy and Crop Science
More specific subject area	Nitrous oxide (N_2_O) fluxes, soil nitrogen contents (nitrate NO_3_^−^ and ammonia NH_4_^+^), aboveground plant nitrogen (N) content, long-term assessment of innovative cropping systems, agricultural practices.
Type of data	Tables and figure
How data were acquired	N_2_O fluxes: calculations based on gas samples collected from static chambers in the field trial. Soil NO_3_^−^ and NH_4_^+^ contents: soil samples collected manually from the field trial; soil N contents determined according to the international standard method NF ISO 14-255 in the laboratory. Aboveground plant N content: analyzed by the Dumas combustion method in the laboratory. Aboveground plant biomass: collected manually from the field trial, oven-dried at 80 °C for 48 h. Yield: collected with a combine harvester from the field trial, oven-dried at 80 °C for 48 h. Agricultural practices: recorded during assessments of the cropping systems in the field trial. Climatic data: collected from an automated meteorological station near the field trial.
Data format	Raw and computation data.
Parameters for data collection	N_2_O fluxes were measured from 2010 to 2016 in two of the four systems. All the other data were collected from 2009 to 2016 in all four cropping systems.
Description of data collection	N_2_O fluxes: HMR process computation of four gas measurements. Mean fluxes, computed from data of three static chambers per plot, in two of the four cropping systems. Monthly measurements, except during August and the winter, with more frequent measurements after periods of fertilizer application. Soil NO_3_^−^ and NH_4_^+^ contents, based on two sets of measurements: (1) a set of data collected at the same time as N_2_O fluxes, at a depth of 0–25 cm or 0–30 cm; (2) a set of data collected during three different periods (at the start and end of winter, post-harvest), at a depth of 0–150 cm. Aboveground plant N content: pool of two or three samples of aboveground plant biomass, depending on the species considered. Aboveground plant biomass: mean of nine to twelve (depending on species) samples (i.e. 1 m² per sample) per plot, collected at maturity. Yield: mean of six samples (i.e. an area of about 140 m² per sample) per plot, harvested at maturity. Agricultural practices: sowing date and density, tillage date and depth, date and amount of mineral N fertilizer spreading, date and type of mechanical weeding, date and type of crop residue management, date of harvest.
Data source location	France, N 48.84°, E 1.95°
Data accessibility	Open Research Data Portal at INRAE; under the CC BY license. https://data.inrae.fr/dataset.xhtml?persistentId=doi:10.15454/EVLGRA. The data tables in the downloaded zip files are provided in both tab and xls formats.
Related research article	C. Colnenne-David, T. Doré, Designing innovative productive cropping systems with quantified and ambitious environmental goals. ``Renewable Agriculture and Food Systems”, 30 (2015) 487–502. https://doi.org/10.1017/s1742170514000313. C. Colnenne-David, G. Grandeau, M-H. Jeuffroy, T. Doré, Ambitious multiple goals for the future of agriculture are unequally achieved by innovative cropping systems, Field Crops Research 210 (2017) 114–128. https://doi.org/10.1016/j.fcr.2017.05.009.

## Value of the Data

• These data were obtained from one of the first long-term field trial (2009-2020) carried out in France, at the AgroParisTech experimental farm at Grignon (France, N 48.84°, E 1.95°). The experiment was focused on the assessment of innovative cropping systems with multiple environmental and production objectives designed to deliver an entire package of ecosystem services. Here, the data were collected for the first eight years of the field assessment.
• These data have already been used to assess the environmental and production performances of the innovative cropping systems [1,2].
• These data can be used as a benchmark for future studies aiming to design new cropping systems to decrease nitrogen (N) losses and improve N management in northern Europe cropping systems.
• These data could be used to improve parameterization and validation, to increase the predictive accuracy of models of N fluxes.
• These data can be used to calculate new indicators based on the measurements of N fluxes.

## 1 Data Set

The open-access research data set is organized into five files of nitrogen (N) data (soil, plant and atmosphere). The raw, descriptive and computed data were collected over the first eight-year period for the four innovative cropping systems assessed in a long-term (2009-–2020) field trial at AgroParisTech experimental farm at Grignon (France, N 48.84°, E 1.95°) [1]. These cropping systems were designed to reach multiple environmental and production objectives and to provide ecosystem services. Practices differ considerably between these four systems, in terms of tillage, N inputs (date and amount), species, potentially modifying soil N content (nitrate NO_3_^−^, ammonia NH_4_^+^), nitrous oxide (N_2_O) fluxes, crop N uptake and yield [2].

The following data are available: (i) N_2_O fluxes, (ii) soil N contents (NO_3_^−^ and NH_4_^+^), (iii) aboveground plant biomass and N content at maturity, and yield, (iv) agricultural practices and (v) climate data.

N_2_O fluxes were calculated for the 2010–2016 period in two cropping systems: the PHEP (productive and high environmental performance) system and the L-GHG (less greenhouse gas emissions) system. All the other data were measured over the 2009–2016 period in all four cropping systems: the PHEP system, the L-GHG system, the No-Pest (no pesticide use) system and the L-EN (less energy consumption) system.

The data sets involved here have already been used: (i) to assess system performances, in terms of greenhouse gas emissions, energy consumption, pesticide use and yield, over the first crop sequence (2009–2014; [2]); (ii) to evaluate and improve a tool for assessing N losses [3,4].

### 1.1 File: 21_06_30_N2O_2010_2016

This file includes data for N_2_O fluxes and soil N (NO_3_^−^ and NH_4_^+^) contents, measured at a depth of 0–25 cm or 0–30 cm, collected at the same time in the PHEP and L-GHG systems over the 2010–2016 period. There are thirteen columns, corresponding to: (1) year of harvest (YYYY), (2) name of cropping system (PHEP and L-GHG); (3) number of replicate (1 to 3); (4) number of plot (1 to 12); (5) species (W and S indicate winter and spring crops, respectively); (6) date of measurement (DD/MM/YYYY); (7) number of the chamber (1 to 3); (8) N_2_O fluxes (g.m^−2^.s^−1^); (9) soil N-NH_4_^+^ content (kgN.ha^−1^); (10) soil N-NO_3_^−^ content (kgN.ha^−1^); (11) soil moisture (((wet soil biomass - dry soil biomass)/ dry soil biomass)*100); (12) soil N-NH_4_^+^ content (mgN.l^−1^); (13) soil N-NO_3_^−^ content (mgN.l^−1^).

### 1.2 File: 21_06_30_Soil_N_2009_2016

This file includes data for soil N (NO_3_^−^ and NH_4_^+^) contents measured at a depth of 0–150 cm, collected at three different time periods (at the start and end of winter, post-harvest), in all four cropping systems over the 2009–2016 period. There are fifteen columns corresponding to: (1) year of harvest (YYYY), (2) name of cropping system (PHEP, L-GHG, L-EN and No-Pest), (3) number of replicate (1 to 3), (4) number of plot (1 to 12), (5) number of sample (1 to 2), (6) species (W and S indicate winter and spring crops, respectively. CC and CI indicate catch crops and cover crops, respectively), (7) period of measurement (BW indicates the beginning of winter, AW stands for after winter and PH for post-harvest), (8) date of measurement (DD/MM/YYYY), (9) soil layer (0–30 cm, 30–60 cm, 60–90 cm, 90–120 cm, 120–150 cm), (10) soil bulk density, (11) soil N-NH_4_^+^ content (kgN.ha^−1^), (12) soil N-NO_3_^−^ content (kgN.ha^−1^), (13) soil moisture (((wet soil biomass - dry soil biomass)/ dry soil biomass)*100), (14) soil N-NH_4_^+^ content (mgN.l^−1^), (15) soil N-NO_3_^−^ content (mgN.l^−1^).

### 1.3 File: 21_06_30_ADM_N_PLANT_2009_2016

This file contains data for the aboveground biomass and N content of the crop measured at maturity in the four cropping systems over the 2009–2016 period. There are eleven columns, corresponding to: (1) year of harvest (YYYY), (2) name of cropping system (PHEP, L-GHG, L-EN and No-Pest), (3) number of replicate (1 to 3), (4) number of plot (1 to 12), (5) number of sample (1 to 12), (6) species (W and S indicate winter and spring crops, respectively), (7) date of measurement (DD/MM/YYYY), (8) development stage (maturity or 8.0 for rapeseed [5]), (9) type of organ collected (seed, stem, stem + pod wall for legumes, straw + rachis for cereals, stalk + cob for maize, straw + panicle for oat or all aboveground organs for rapeseed), (10) crop aboveground biomass (t. ha^−1^), (11) crop aboveground N content (% of dry matter).

### 1.4 File: 21_06_30_ITK_2009_2016

This file contains data relating to agronomic practices and yield collected for the four cropping systems over the 2009–2016 period. There are nineteen columns, corresponding to: (1) year of harvest (YYYY), (2) name of cropping system (PHEP, L-GHG, L-EN and No-Pest), (3) number of replicate (1 to 3), (4) number of plot (1 to 12), (5) species (W and S indicate winter and spring crops, respectively), (6) crop characteristics (cover crop or catch crop species), (7) description of soil: bare soil (yes or no), (8) date of agricultural practice (DD/MM/YYYY), (9) agricultural practice (sowing, tillage, mineral fertilization, mechanical weeding, harvest), (10) depth of plowing (cm), (11) sowing density (kg.ha^−1^), (12) variety, (13) amount of N applied (kg.ha^−1^), (14) type of N fertilizer (mineral or organic), (15) name and N concentration of the fertilizer (%), (16) fertilizer location (surface or incorporated into the soil), (17) status of crop residue (stubble left in place or exported), (18) mean yield (tonnes of dry matter.ha^−1^), (19) standard deviation of yield (tonnes of dry matter.ha^−1^).

### 1.5 File: 21_06_30_CLIMATIC_2009_2016

This file contains climate data over the 2009–2016 period. There are four columns, corresponding to: (1) date of measurement (DD/MM/YYYY), (2) mean daily temperature (°C), (3) daily rainfall (mm), (4) mean daily soil temperature at 10 cm below the surface (°C).

## 2 Experimental Design

### 2.1 Field experiment site

The experiment took place at the AgroParisTech experimental farm at Grignon, in the Ile-de-France region (N 48.84°, E 1.95°: France). The field was characterized by a deep and homogeneous loamy clay soil (haplic luvisol according to the FAO classification [6]). The mean soil characteristics of the plowed layer (0–25 cm) in 2009 were as follows: clay content = 20.6 g.kg^−1^, silt content = 71.9 g.kg^−1^, sand content = 7.4 g.kg^−1^, bulk density = 1.4, CEC = 11.5 cmol+ .kg^−1^ and carbon content = 15.9 g.kg^−1^. The C/N ratio was 12.4 and the pH was 6.9 (further details are provided for each plot, in Table 1). The experimental field was flat, with a water table more than 2 m below the surface and an available water storage capacity of about 175 mm. The trial took place in an area with an oceanic climate. Over a period of 20 years, mean rainfall was 650 mm per year and mean daily temperature was 12.5 °C. The previous crop, in 2008, was winter barley. The field was plowed to a depth of 30 cm after the barley harvest. After six years of the experiment, various tillage practices in the different systems (*i.e.* no-till practices in both the L-EN and L-GHG systems; four plowings over six years in the No-Pest system) had induced changes in bulk density requiring a second measurement in 2014.

**Table 1:** Physicochemical properties of the soil for each plot (layer = 0–25 cm, in 2009). Sampling method: plots were divided into four subplots. For each subplot, we collected, pooled and analyzed seven samples (clay (g.kg^−1^), silt (g.kg^−1^) and sand (g.kg^−1^): NF ISO 31-107; organic carbon (g.kg^−1^): NF ISO 10-694; total nitrogen (g.kg^−1^): NF ISO 13-878; CaCO_3_ (g.kg^−1^): NF ISO 10-693; pH: NF ISO 10-390; CEC (cmol+ .kg^−1^): Metson method, NF ISO 31-130N). Bulk density (g.cm^−3^) was measured with a steel cylinder with a cross-sectional area of 98 cm^3^ inserted vertically into the soil (0–30 cm). The plot values provided are the means of the four subplot results. PHEP (productive with high environmental performance), L-GHG (low greenhouse gas emissions), L-EN (low energy consumption), No-Pest (no pesticide use). Rep = replicate. Org Carb = organic carbon. Tot N = total nitrogen. BD = bulk density. MD = missing data.

Cropping			Clay	Silt	Sand	Org Carb	Tot N	CaCO_3_		CEC	BD 2009	BD 2014
system	Rep	Plot	(g.kg^−1^)	(g.kg^−1^)	(g.kg^−1^)	(g.kg^−1^)	(g.kg^−1^)	(g.kg^−1^)	pH	(cmol+.kg^−1^)	(g.cm^−3^)	(g.cm^−3^)
PHEP	1	1	214	713	73	15.8	1.52	1.05	7.35	12.0	1.42	1.48
PHEP	2	7	222	705	73	13.5	1.30	<1.00	6.78	12.0	1.43	1.47
PHEP	3	10	201	733	66	14.5	1.34	<1.00	6.60	11.3	1.47	1.44
L-GHG	1	4	201	717	82	17.1	1.56	2.02	7.54	12.0	1.40	1.44
L-GHG	2	6	226	710	64	14.0	1.38	<1.00	6.76	12.0	1.43	1.55
L-GHG	3	9	201	727	72	13.9	1.36	<1.00	6.39	11.5	1.47	1.51
L-EN	1	3	203	715	82	15.7	1.50	7.23	7.73	11.6	MD	1.43
L-EN	2	5	196	730	74	13.6	1.23	<1.00	7.00	10.5	MD	1.55
L-EN	3	12	183	678	74	13.8	1.28	<1.00	6.58	10.9	MD	1.52
No-Pest	1	2	218	703	79	15.8	1.52	6.54	7.76	12.0	MD	1.38
No-Pest	2	8	213	711	76	14.7	1.25	<1.00	6.41	11.4	MD	1.37
No-Pest	3	11	201	729	77	15.9	1.39	<1.00	6.25	11.0	MD	1.41

### 2.2 Experimental design

**Fig. 1:**
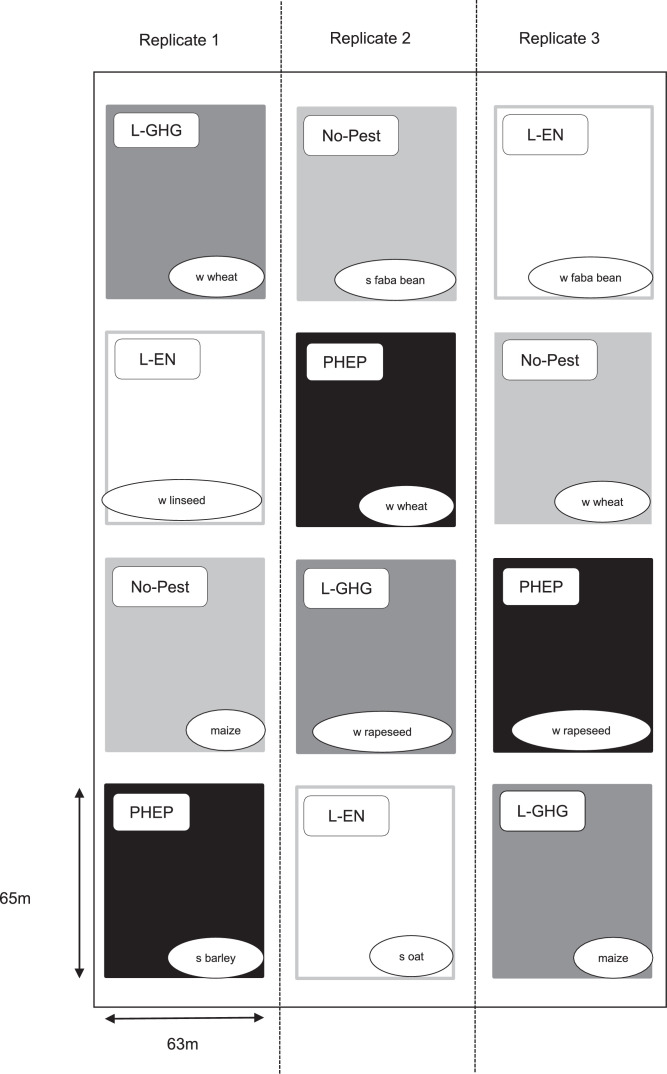
Experimental design in 2009, with four cropping systems and three replicates, located at the AgroParisTech experimental farm (France, N 48.84°, E 1.95°). The crops sown in 2009 on each of the 12 plots are indicated. Cropping systems are as follows: PHEP (productive with high environmental performance), L-GHG (low greenhouse gas emissions), L-EN (low energy consumption), No-Pest (no pesticide use). W and S indicate winter and spring crops, respectively.

The trial covered a total area of 6.2 ha, divided into three replicates (Fig. 1). Each replicate was split into four wide plots, each dedicated to one of the four cropping systems. In the three replicates of each cropping system, three different crops from the crop sequence were sown in each year (e.g. in 2009, winter wheat, winter rapeseed and maize were sown in the three replicates of the L-GHG system; see [2] for more details). The three replicates were managed according to similar decision rules, resulting in different practices (*e.g.* date and amount of N fertilizer applications) due to environmental factors and working organization constraints [7]. Farm machinery was used, as the area devoted to this experiment was almost 4000 m² per replicate.

### 2.3 Innovative cropping systems

The PHEP system was designed to minimize environmental impact: (i) cover crops were sown before each spring species to decrease nitrate losses; (ii) pesticide uses were lessened by increasing crop diversity, lengthening the crop sequence and sowing highly resistant varieties; (iii) energy consumption was reduced by allowing plowing only once in the crop sequence, and N fertilizer amounts spread were decreased by the incorporation of legumes into the crop sequence. This cropping system was also designed to reach the maximum yield given the environmental targets, as described in [1]. This cropping system, designed without major environmental constraint, was used as the reference system for comparisons with the other systems.

In the L-GHG system, greenhouse gas emissions were limited by increasing carbon sequestration in the soil (i.e. producing large amounts of residues from both the main crop and catch crops without tillage) and N_2_O emissions were decreased by using appropriate decision rules to prevent N fertilizer application in climatic conditions favoring N_2_O emissions.

The L-EN system was designed to reduce both direct and indirect energy consumption. Plowing was prohibited, direct sowing was implemented, the amount of N fertilizer applied was decreased by sowing many legumes and species with high N use efficiency, and target yield were decreased.

For the No-Pest cropping system, the no-pesticide constraint was satisfied by including a wide range of species (*e.g.* hemp), the use of highly resistant varieties or species mixtures, a wide diversity of sowing dates, regular tillage and mechanical weeding to control weeds.

These three cropping systems, each designed with a major environmental constraint, were also required to meet the same environmental and yield goals as achieved by the PHEP system. During the design step, the constraints and targets were prioritized as follows: the environmental constraint had to be satisfied first, the set of other environmental targets then had to be reached and, finally, yield had to be maximized.

## 3 Materials and Methods

### 3.1 N_2_O fluxes

N_2_O fluxes were calculated for each of the three replicates of the PHEP and L-GHG systems. Data were collected monthly, except during the winter (from November to March) and August. Additional measurements were carried out during specific periods: four measurements were performed over a two-week period after each N fertilizer application; two measurements were made after the faba bean harvest if a significant rainfall event occurred (>10 mm). For each plot, N_2_O emissions were measured manually, with three static chambers inserted into the soil at a depth of 10 cm after sowing and left in place until harvest. Each chamber covered a surface area of 0.25 m^2^ and the top of the chamber was 0.2 m above the soil surface. N_2_O emissions were measured between 10 a.m. and 3 p.m. (local time) at each sampling date, with the same order used for monitoring on each plot. The chambers were closed for 45 min, during which the headspace air was sampled four times (0, 15, 30 and 45 min after closure). Gas samples (20 mL) were collected in a syringe and immediately injected into pre-evacuated 12 mL glass vials (Exetainers, Labco, UK). These vials were stored in the dark at room temperature until laboratory analyses. N_2_O concentrations were analyzed with a gas chromatograph equipped with an electron capture detector (GC-ECD; Model 3800, Varian Inc., CA, USA; see more details in [8]). N_2_O fluxes were calculated with the HMR model (i.e. R statistical core software [9]), from four successive measurements of the N_2_O content of gas samples.

### 3.2 Soil N contents (NO_3_^−^ and NH_4_^+^)

Two sets of soil N content data were managed and are provided in two different files:

Set 1: measurements performed at the same time as those for N_2_O fluxes. Three randomized soil samples were collected close to the static chambers, at the depth either 0–25 cm or 0–30 cm, and pooled (data provided in file 21_06_30_N2O_2010_2016, columns 9, 10, 12 and 13).
Set 2: measurements performed at three different periods over the year (i.e. at the beginning of winter (BW) around November 15, after winter (AW) around February 15, and about eight days post harvesting (PH) of the main crop). Six samples were collected from five layers (0–30 cm, 30–60 cm, 60–90 cm, 90–120 cm, and 120–150 cm) in each plot. Three samples from each layer were pooled to generate two soil samples per layer for each plot (data are in file 21_06_30_Soil_N_2009_2016, columns 11, 12, 14 and 15).

These two groups of measurements were managed in the same way. Soil samples were collected manually with an auger and stored in a cold box (4 °C) until analysis. Water content was measured gravimetrically, according to the international standard method (NF ISO 11-465). From a soil sample (50 g), soil inorganic N was extracted in potassium chloride solution (77 g.l^−1^) with the aid of a magnetic stirrer for 30 min. After 30 min of decantation, the suspended matter has settled and the supernatant was collected. Supernatant aliquots were sent to the analytical laboratory. NO_3_^−^ was determined by reaction with N-(1-naphthyl) dichloride diamine ethylene, and NH_4_^+^ was determined by reaction with sodium dichloro-isocyanurate and sodium salicylate. NO_3_^−^ and NH_4_^+^ contents were analyzed in an aliquot of the extracts obtained, by colorimetry (absorbance measured at 550 nm and at 630 nm, respectively; international standard method: NF ISO 14-255). Results were expressed both in kgN per hectare and in mgN per liter.

### 3.3 Aboveground biomass and N content at maturity

Depending on species, we collected nine to twelve samples (i.e. 1 m² per sample) per plot at maturity, except for winter rapeseed, for which samples were collected at stage 8.0 [5]. Due to the high aboveground biomass for maize, we divided each sample into two subsamples (*A* and *B*) in all years except in 2009. Seeds were separated from the vegetative parts of the plant (straw and pod walls for legumes, straw and rachis for cereals, straw and panicles for oat, stalk and cobs for maize), except for winter rapeseed, for which all aboveground parts (stems, pods and green seeds) were pooled. All samples were oven-dried at 80 °C for 48 h. For analyses of N content, we pooled two or three samples, depending on species, which were then ground and analyzed by the Dumas combustion method [10].

### 3.4 Yield

Yield (mean and standard deviation) were calculated on the basis of six samples (i.e. an area of about 140 m² per sample, depending on the length of the plot harvested) collected at maturity, with a combine harvester, from each plot. Yield unit was tonne of dry matter per hectare.

### 3.5 Agricultural practices in the innovative cropping systems

The management of the four cropping systems has been described in detail in [2]. The crop sequences included five crops for the PHEP and L-EN systems, and six crops for the No-Pest and L-GHG systems. The species sown in each replicate of each system over the 2009–2016 period are detailed in Table 2. All agricultural practices were recorded continuously and only those linked to N fluxes are reported in the file: (i) date and density of sowing, (ii) date and depth of tillage, (iii) date and amount of mineral N fertilizer applied, (iv) date and type of mechanical weeding, and (v) date and type of crop residue management.

**Table 2:** Crop sequences of the four cropping systems over the 2009–2016 period. PHEP: productive with high environmental performance; L-GHG: low greenhouse gas emissions; L-EN: low energy consumption; No-Pest: no pesticide use. Rep = replicate. W and S indicate winter and spring crops, respectively. W wheat* and W rapeseed*: intercropping of a legume with winter wheat and winter rapeseed, respectively. For crop sequences including two W wheat crops, W wheat1 indicates a W wheat crop sown after a legume species, W wheat2 indicates a W wheat crop sown after a non-legume species. Table modified from [2].

Cropping system	Rep	Plot	2009	2010	2011	2012	2013	2014	2015	2016
PHEP	1	1	S barley	W faba bean	W wheat1	W rapeseed	W wheat2	S barley	W faba bean	W wheat1
PHEP	2	7	W wheat2	S barley	W faba bean	W wheat1	W rapeseed	W wheat2	S barley	W faba bean
PHEP	3	10	W rapeseed	W wheat2	S barley	W faba bean	W wheat1	W rapeseed	W wheat2	S barley
L-GHG	1	4	W wheat	W barley	Maize	Triticale	S faba bean	W rapeseed	W wheat	Soybean
L-GHG	2	6	W rapeseed	W wheat	W barley	Maize	Triticale	S faba bean	W rapeseed	W wheat
L-GHG	3	9	Maize	Triticale	S faba bean	W rapeseed	W wheat	W barley	Maize	W peat
L-EN	1	3	W linseed	W wheat2*	S oat	W faba bean	W wheat1	W rapeseed*	W wheat2	S oat
L-EN	2	5	S oat	W faba bean	W wheat1	W linseed	W wheat2*	S oat	Soy bean	W wheat1*
L-EN	3	12	W faba bean	W wheat1	W linseed	W wheat2*	S oat	Soy bean	W wheat*	W rapeseed*
No-Pest	1	2	Maize	W wheat2	S faba bean	W wheat1	Hemp	Triticale	Maize	W wheat2
No-Pest	2	8	S faba bean	W wheat1	Hemp	Triticale	Maize	W wheat2	S faba bean	W wheat1
No-Pest	3	11	W wheat2	S faba bean	W wheat1	Hemp	Triticale	Maize	W wheat2	S faba bean

### 3.6 Climatic data

Mean daily temperatures (°C) and daily rainfalls (mm) data were collected from an automated INRAE meteorological station (no. 78615002: latitude 48.838°N, longitude 1.953°E, elevation: 125 m) located 150 m from the trial.

## CRediT Author Statement

**Caroline Colnenne-David:** Conceptualization, Methodology, Validation, Formal analysis, Resources, Writing – original draft, Writing – review & editing, Project administration, Funding acquisition, Supervision; **Gilles Grandeau:** Resources; **Marie-Hélène Jeuffroy:** Conceptualization, Writing – original draft, Funding acquisition; **Thierry Doré:** Conceptualization, Writing – original draft, Writing – review & editing, Funding acquisition, Supervision.

## Declaration of Competing Interest

The authors declare that they have no known competing financial interests or personal relationships that have or could be perceived to have influenced the work reported in this article.

## Appendix Supplementary Materials

Image, application 1

Image, application 2

Image, application 3

Image, application 4

Image, application 5
